# Supplementary material for: Comparison of Alternative Splicing Landscapes Revealed by Long-Read Sequencing in Hepatocyte-Derived HepG2 and Huh7 Cultured Cells and Human Liver Tissue
Source: Biology (Basel). 2023 Dec 6;12(12):1494. doi: 10.3390/biology12121494 (PMC10740679; doi:10.3390/biology12121494)
Supplement: Supplementary file 1 [file biology-12-01494-s001.zip › Figure S1.pdf]

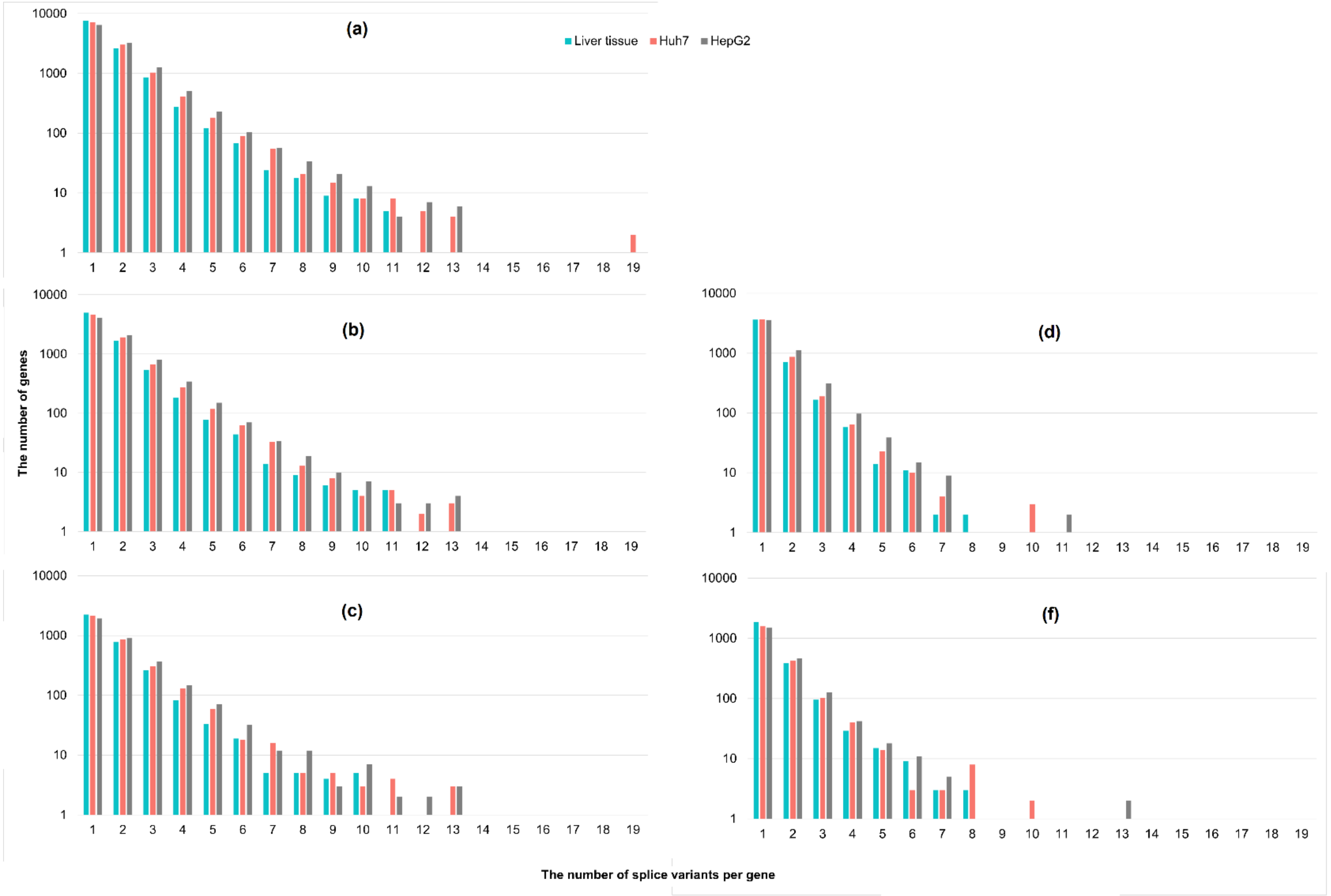

Figure S1. The distributions of the number of genes by the number of splice variant assigned to a gene. **(a)** all (14427) detected genes; **(b)** 9235 genes randomly selected out of 14427 detected genes; **(c)** 4236 genes randomly selected out of 14427 detected genes; **(d)** 9235 genes with phenotype-specific expression; **c)** 4236 genes with phenotype-specific expression of one or two splice variants.
